# Longitudinal assessment of grip strength using bulb dynamometer in Duchenne Muscular Dystrophy

**DOI:** 10.1590/bjpt-rbf.2014.0031

**Published:** 2014

**Authors:** Tatiana M. Pizzato, Cyntia R. J. A. Baptista, Mariana A. Souza, Michelle M. B. Benedicto, Edson Z. Martinez, Ana C. Mattiello-Sverzut

**Affiliations:** 1 Department of Biomechanics, Medicine and Rehabilitation of the Locomotor Apparatus, Faculty of Medicine of Ribeirão Preto (FMRP), Universidade de São Paulo (USP), Ribeirão Preto, SP, Brazil; 2 Department of Public Health, Faculty of Medicine of Ribeirão Preto (FMRP), USP, Ribeirão Preto, SP, Brazil

**Keywords:** muscular dystrophy, skeletal muscle, muscle strength, dynamometer, physical therapy

## Abstract

**BACKGROUND:**

Grip strength is used to infer functional status in several pathological conditions, and the hand dynamometer has been used to estimate performance in other areas. However, this relationship is controversial in neuromuscular diseases and studies with the bulb dynamometer comparing healthy children and children with Duchenne Muscular Dystrophy (DMD) are limited.

**OBJECTIVE:**

The evolution of grip strength and the magnitude of weakness were examined in boys with DMD compared to healthy boys. The functional data of the DMD boys were correlated with grip strength.

**METHOD:**

Grip strength was recorded in 18 ambulant boys with DMD (Duchenne Group, DG) aged 4 to 13 years (mean 7.4±2.1) and 150 healthy volunteers (Control Group, CG) age-matched using a bulb dynamometer (North Coast- NC70154). The follow-up of the DG was 6 to 33 months (3-12 sessions), and functional performance was verified using the Vignos scale.

**RESULTS:**

There was no difference between grip strength obtained by the dominant and non-dominant side for both groups. Grip strength increased in the CG with chronological age while the DG remained stable or decreased. The comparison between groups showed significant difference in grip strength, with CG values higher than DG values (confidence interval of 95%). In summary, there was an increment in the differences between the groups with increasing age. Participants with 24 months or more of follow-up showed a progression of weakness as well as maintained Vignos scores.

**CONCLUSIONS:**

The amplitude of weakness increased with age in the DG. The bulb dynamometer detected the progression of muscular weakness. Functional performance remained virtually unchanged in spite of the increase in weakness.

## Introduction

The main clinical signs of Duchenne Muscular Dystrophy (DMD) are muscular weakness that begins proximally and spreads to the extremities, abnormal walking, frequent falls, and difficulties in climbing stairs^1^ ^-^ ^3^. Functional changes related to upper limb muscular weakness normally appear 2 or 3 years after the onset of pelvic girdle signs^4^, and transversal studies are limited to distinguish changes in strength due to the development or slow advance of diseases such as DMD. According to Stuberg and Metcalf^5^, muscular weakness in DMD becomes apparent around age 6 to 8 and upper limb weakness usually emerges around age 8 to 11.

In a conventional clinical evaluation, the measurement of muscular strength infers the integrity of the neuromuscular system and allows the establishment of correlations with the individual's quality of life. For this evaluation and measurements of muscular strength in different body segments, the common procedures are a manual muscle test and/or analysis by dynamometry. To analyze grip strength, several studies used the mechanical or electronic dynamometer in association with the manual muscle test^6^ ^-^ ^8^. The authors report that the procedures generate accurate information on the progression of specific muscle group weakness in children with DMD and help in the choice of the most adequate therapeutic interventions. Muscular fiber architecture, age, gender, muscle size and length at contraction, muscular average, and contraction velocity as well as the child's emotional and cognitive stages are some of the factors involved in the performance of muscular strength^9^. Issues such as calibration and the shape of the dynamometer's handle have an influence on measurements^10^.

Quantitative muscle tests (QMTs) have been employed in studies on neuromuscular diseases^6^ ^,^ ^11^ ^-^ ^14^. Some of the devices, such as the Jamar dynamometer and the strain gauge hand dynamometer, were considered predictors of impairment including the loss of independent ambulation^5^. However, Escolar et al.^11^ point out that QMTs have not been confirmed a measure of strength in large multicenter studies in children with neuromuscular diseases, showing low specificity for DMD due to scarce longitudinal studies in healthy and DMD children. Studies with the hand held dynamometer in normal individuals infer functional activity of the lower limbs^15^ ^,^ ^16^. However, the relationship between grip strength and functional activities in neuromuscular disorders such as DMD was poor, according to Vandervelde et al.^14^. Longitudinal studies with bulb dynamometer measurements comparing healthy and DMD children were not found, and the relationship between motor impairment and functional performance remains to be explored.

The main objective of this observational study was to follow the evolution of grip strength in participants with DMD using the bulb dynamometer and to examine the range of the weakness compared with data from healthy participants. Additionally, functional data obtained from the DMD participants using the Vignos scale were correlated with grip strength.

## Method

### Participants

### Patients

Eighteen DMD patients (n=18) aged 4 to 13 years (mean 7.4±2.1) recruited at the Neuromuscular Outpatient Clinic of the University Hospital of the Faculty of Medicine of Ribeirão Preto, Universidade de São Paulo (HCFMRP-USP), Ribeirão Preto, SP, Brazil, participated in the study and composed the Duchenne group (DG). The inclusion criteria were diagnosis of DMD, community ambulation, cognitive ability to understand the required task of exercising grip strength, and attendance at a minimum of 3 evaluation sessions (9 month follow-up).

### Controls

One hundred and fifty healthy volunteers with compatible age constituted the control group (CG). Inclusion criteria for the CG were absence of any musculoskeletal, neuromuscular or degenerative disease. One grip strength trial was conducted for each volunteer using the same test and under the same conditions as the Duchenne participants.

The study was approved by the Research Ethics Committee of HCFMRP-USP (protocol no. 6990/07). All parents/guardians signed an informed consent form.

### Materials

The North Coast bulb dynamometer (NC 70154) was used for the quantitative measurements in a series of three trials. The equipment has a high-impact plastic measuring device and a soft rubber bulb with a 13 cm circumference. For the dynamometer calibration, the red pointer was positioned at the zero mark before each trial. Measures were expressed in fractions of pounds/per square inch (psi) and range between 0 and 30 psi.

### Design and procedure

The DG data records were obtained at the Rehabilitation Center of HCFMRP at 3-month intervals. For the DG, the evaluation was conducted for at least 9 months (3 evaluation sessions) and at most 33 months (12 evaluation sessions). For the CG participants, only one evaluation was conducted in the school environment and age-matched with the DG, since the longitudinal development of strength was not the focus of this study.

The functional performance of the DG was evaluated using the Vignos scale over the course of the study. This is a 9-point scale that describes a variety of activities relating to the lower limbs, with lower scores indicating better performance.

Evaluation sessions were standardized and conducted by the same examiner in three trials for each hand. Before each trial, the calibrated dynamometer had the pointer set to zero and the child was seated comfortably on a bench without support for arms and legs, adducted arm, 90^o^ flexed elbow, forearm and wrist in the neutral position, according to recommendations by the American Society of Hand Therapists^17^. After a demonstration, the child was instructed to hold the device and in a comfortable manner squeeze the bulb as hard as they could for 5 seconds. For each participant, the mean of three bilateral measurements was considered for each evaluation session. The interrater reliability of the bulb dynamometer has been previously tested, and it was shown to be a reliable instrument to evaluate muscular strength in healthy children. A high agreement index was obtained for the three measurements in the same evaluation session. The values in the first evaluation, ICC= .82 to .83 for the right hand and ICC=.87 to .88 for the left hand, improved in the three next evaluations (75%), ICC=.93 to .95, right hand and ICC=.92 to .95, left hand, again with excellent agreement.

### Statistical analysis

Data were assessed using descriptive statistics (mean, standard deviation) to test differences between the DG and CG, and linear regression for mixed effects was applied to grip strength. Spearman's correlation coefficient was used to assess the relationship between grip strength and functional performance (Vignos scale score).

## Results

### Characterization of participants

### General analysis

The main clinical data of the DG are shown in Table 1. The youngest participant was 49 months and the oldest, 156 months. In the beginning of the study, functional status measured by the Vignos scale indicated that 83% of patients scored 1 to 3, meaning that they still could walk and climb stairs. All DMD boys were submitted to corticotherapy, with the exception of participant F. According to his parents, he wore an ankle foot orthosis (night or daily regime) and went to physiotherapy/hydrotherapy at least once a week. The follow-up for each participant is shown in Table 1.

**Table 1.** Characteristics of the DMD group participants according to age, functional status, drug therapy, use of orthosis, physiotherapy/ hydrotherapy intervention, and duration of follow-up.

Participant	Age (months)	Initial Functional Status (Vignos)	Final Functional Status (Vignos)	Drug therapy	Orthosis	Physiotherapy/hydrotherapy (sessions per week)	Follow -up (month)
A	49	1	2	Predsim	none	3/0	6
B	57	4	5	Prednisone	rigid AFO/ night splint	2/1	6
C	60	2	2	Deflazacort	night/ day	2/0	9
D	65	2	2	Meticorten	rigid AFO night	1/1	30
E	68	3	4	Predsim	rigid AFO night splint	2/0	21
F	73	2	3	Refused	rigid AFO night	1/1	12
G	80	3	5	Prednisone	rigid AFO/ night splint	2/0	12
H	85	1	2	Prednisone	night/day	3/0	6
I	87	1	2	Prednisone	night/day	2/1	30
J	88	2	2	Deflazacort	night/ day	3/0	24
K	90	3	2	Prednisone	night/ day	2/0	33
L	93	4	5	Prednisone	rigid AFO night	2/1	12
M	97	3	4	Prednisone	night/ day	2/0	33
N	106	3	4	Deflazacort	night/ day	2/2	15
O	106	1	2	Deflazacort	rigid AFO night	2/1	15
P	108	1	3	Corticorten	rigid AFO night	1/1	27
Q	130	2	2	Deflazacort	none	2/0	24
R	156	5	5	Budecort	rigid AFO/ night	2/0	9

The participants of the CG were boys of 49 to 156 months years of age (mean of 89 months). All participants were non-athletes (they could participate in up to 3 physical activities per week, in different athletic modalities) and attended regular primary schools.

Based on a confidence interval of 95% (CI95%), the comparison of the dominant and non-dominant grip strength considering the DG and CG indicated no significant difference between them (Table 2). However, the comparison of grip strength between groups (DG and CG), considering the dominant and non-dominant hand, indicated the CG obtained higher values than the DG (Table 2). Note that the mean values for the DG grip strength and CI95% remain quite stable regardless of age. Furthermore, these CG data suffered increment with chronological age. In summary, there was an increment in the differences between the groups as shown in the column "Difference" in Table 2.

**Table 2.** Mean of grip strength and differences between the Duchenne group and the control group according to confidence interval (CI95%), considering age and hand dominance.

Age	Duchenne Group				Control Group				Difference		
(months)	Number of Evaluations	Mean	CI 95%		Number of Evaluations	Mean	CI 95%		Mean	CI 95%	
Dominant hand											
Up to 80	36	3.44	(2.55	4.38)	45	4.36	(3.51	5.21)	0.92	(-0.43	2.11)
81 to 90	39	3.30	(2.40	4.23)	63	5.45	(4.74	6.16)	2.15	(0.99	3.33)
91 to 100	63	3.41	(2.53	4.31)	45	6.18	(5.32	7.01)	2.76	(1.51	4.01)
101 to 110	57	3.39	(2.48	4.34)	54	6.22	(5.41	7.02)	2.82	(1.66	4.03)
111 to 120	57	3.33	(2.47	4.25)	0	-			-		
121 to 130	33	3.16	(2.22	4.09)	63	6.86	(6.12	7.60)	3.69	(2.50	4.91)
131 to 140	27	3.49	(2.57	4.41)	51	7.93	(7.10	8.76)	4.43	(3.15	5.68)
141 to 150	9	3.77	(2.78	4.80)	57	9.21	(8.49	9.99)	5.43	(4.18	6.70)
Up to 150	18	3.13	(2.04	4.17)	66	11.36	(10.6	12.08)	8.22	(6.83	9.48)
Non-dominant hand											
Up to 80	36	3.42	(2.53	4.38)	45	4.07	(3.21	4.88)	0.65	(-0.69	1.82)
81 to 90	39	3.28	(2.40	4.23)	63	5.29	(4.59	6.00)	2.01	(0.88	3.15)
91 to 100	63	3.36	(2.49	4.29)	45	5.78	(4.91	6.61)	2.41	(1.14	3.64)
101 to 110	57	3.42	(2.54	4.32)	54	5.76	(4.93	6.58)	2.34	(1.17	3.52)
111 to 120	57	3.22	(2.37	4.12)	0	-			-		
121 to 130	33	3.27	(2.37	4.17)	63	6.67	(5.95	7.39)	3.40	(2.24	4.57)
131 to 140	27	3.21	(2.29	4.15)	51	7.18	(6.29	8.01)	3.96	(2.65	5.22)
141 to 150	9	3.26	(2.21	4.35)	57	8.45	(7.70	9.23)	5.18	(3.91	6.45)
Up to 150	18	3.08	(2.04	4.09)	66	10.72	(9.99	11.45)	7.64	(6.39	8.93)

The relationship between grip strength and functional capacity (Vignos scale) of the participants of the DG, tested using Spearman's coefficient (rho), indicated a poor inverse correlation between the Vignos scores and grip strength obtained at baseline (rho=-0.3) and at the end of the study (rho=-0.5).

### Individual analysis

To analyze the individual behavior of the participants of the DG, we used the mean of the dominant grip strength for each session obtained during the experimental period (Table 3). The youngest participants of the DG (A, B, and C) had a brief follow up, their grip strength values were close to those of the healthy age-matched participants, and the Vignos score was almost the same. Participants D, I, J, M, P, and Q had a follow up of 24 months or more and, in general, their grip strength decreased or became stable (Table 3). For example, participant Q (the oldest participant followed up for 24 months) showed a slow progression of weakness (4.4 to 3.3 psi - first to last assessment) as well as maintained Vignos scores. Participants K and M, who were followed up for 33 months, showed oscillations in grip strength throughout the evaluations, but the initial and final values remained at about 3 psi. Participant I (72 months old) was one exception in the DG, presenting an increment in grip strength after 30 months (2.5 psi to 3.7 psi), but without reaching normal values.

**Table 3.** Mean of individual dominant grip strength obtained in each session over the course of the study.

Participant (age in months at the time of admission)	Mean of grip strength (psi) in each session											
A (49)	2.3	2.2	2.5									
B (57)	0.5	1.5	2.0									
C (60)	3.8	3.8	3.8	4.0								
D (65)	2.5	3.0	3.3	3.8	2.0	2.2	2.7	2.5	3.5	2.8	2.5	
E (68)	2.5	2.7	3.0	2.7	2.2	1.7	2.0	2.2				
F (73)	2.3	1.3	2.0	3.0	2.5							
G (80)	3.7	2.0	3.3	2.7	2.0							
H (85)	3.5	3.0	2.7									
I (87)	2.5	3.3	3.5	4.5	4.3	3.3	4.0	4.2	4.2	4.0	3.7	
J (88)	5.8	5.3	5.5	5.5	3.2	5.3	5.5	6.0	5.7			
K (90)	3.0	4.3	3.8	4.2	4.2	4.2	3.2	3.7	3.5	3.8	3.2	3.2
L (93)	3.3	3.8	2.8	2.8	3.3							
M (97)	3.2	3.5	4.5	4.7	5.0	3.0	2.3	3.0	3.5	3.5	3.2	3.2
N (106)	3.5	2.8	3.8	3.5	2.3	2.0						
O (106)	3.7	1.8	2.8	3.0	4.0	3.7						
P (108)	4.5	5.5	5.5	5.2	3.3	5.2	5.5	5.7	4.7	4.8		
Q (130)	4.7	3.0	2.7	3.0	3.3	3.7	3.8	3.0	3.3			
R (156)	2.8	3.0	3.3	2.5								

## Discussion

This study used bulb dynamometer data to analyze the amplitude of grip weakness in ambulant children with DMD compared to healthy children in a maximum period of 33 months. The data acquired here and clinically employed suggests that grip strength measured by the bulb dynamometer is a useful tool to detect the evolution of the disease and it could be introduced in routine physical examinations or even serve as a measure of the effect of several interventions. However, it seems that general functional performance could not be estimated based on grip strength, as suggested for the healthy population. Obviously, grip strength deficit could be useful to show its specific impact on upper limb activities during the evolution of the disease.

Variability in individual features, clinical presentation, and exposure to different therapeutic interventions could be a source of bias when analyzing the progression of weakness in our participants, however it is not possible to control such interferences. Data about the treatments and clinical conditions were included here to show that there were no discrepancies between participants, with the exception of participant F who refused medication. Based on that data, we assume that the participants were exposed to similar conditions with regard to factors involved in the progression of the disease. The analysis of the effects of these interventions is out of the scope of this study.

Most of the data in the literature demonstrate a linear decrease in muscle strength in the DG^18^ ^,^ ^19^, while other studies showed minimal changes until age 8 or even an increase in strength resulting from development. Compromised grip strength is considered the last symptom of DMD^19^ ^,^ ^20^. For Stuberg and Metcalf^5^, muscular weakness in DMD becomes apparent around age 6 to 8 with upper limbs showing this symptom 2 or 3 years after, therefore, upper limb weakness should emerge around age 8 to 11. However, our data showed evident weakness around the age of 6, indicating that weakness was advanced in our sample. These data serve as advice and warrant further investigation into the case of our participants to explain the early onset of upper limb weakness.

In McDonald et al.^19^, most of the muscle groups measured by quantitative methods showed that DMD strength was 35-50% of normal values. Considering that the distal muscles are affected later, we hypothesized that the magnitude of differences between normal and DMD grip strength would be lower than the findings presented by McDonald et al.^19^. However, our data demonstrated that even DMD children at the age of 120 months presented with grip strength 50% below normal, as already stated by Cech^7^.

The progress of this disease determines the negative impact on strength. However, the real impact of this disease during a child's development has not been objectively published. This longitudinal follow up showed that the young boys (80 months) presented a decrement in strength when compared with normal children of the same age. Similar data about impaired muscular strength in neuromuscular diseases have been reported^14^ ^,^ ^21^ ^,^ ^22^, but without considering longitudinal analysis of the upper limbs or using the bulb dynamometer. In a transversal study, Mattar and Sobreira^22^ evaluated 40 DMD patients and detected an ascending curve of the compromised grip strength starting at the onset of the disease, followed by a descending curve in older patients. Burns et al.^21^reported that children with Charcot-Marie-Tooth Type 1 showed decreased grip strength measured by the hand dynamometer between the ages of 2 and 16.

The data of our CG was representative of normal grip strength when compared with the results found by Molennar et al.^23^. Therefore, based on our comparisons, the DG presented a decrease in muscular strength in all ages, although the differences between healthy and DMD boys was exacerbated from age 10 (120 months) onwards.

The aim of the present study was not a full evaluation of the evolution of the disease since it involves several aspects besides strength measurement. Grip weakness could be detected even in the early stages of the disease, but functional performance did not change significantly. The initial and final Vignos scores tended to oscillate only one point, with exception of participants G and P who passed from 3 to 5 and 1 to 3, respectively. The poor inverse correlation between grip strength and the Vignos scores confirms these findings and agrees with Vandervelde et al.^14^, who found correlations between functional limitation and grip strength only in patients with proximal neuromuscular disorders and a higher correlation in patients with spinal muscular atrophy (r=.86 and .82), followed by DMD, Becker muscular dystrophy, and muscular limb-girdle dystrophy (r=.53 to .59).

Thus, the combination of grip strength with functional scales, which provide information about the interaction between strength, activities of daily living (ADLs), analysis and control of movements, muscular fatigue, and auxiliary devices, is relevant and adds real data about the patient. Nevertheless, for DMD, the direct relationship between functional performance and grip strength was not strong.

Some limitations in this study are related to the lack of investigation of the specific genetic mutation in dystrophin for our sample. Also, the number of follow-up sessions was different according to the insertion of the participant in the study, a fact that limited the observation of grip strength evolution for some participants. Furthermore the selection of only ambulant boys drastically reduced the number of participants, as they were recruited from a tertiary care center. Therefore, new longitudinal studies are needed to confirm the results reported here.

## Conclusion

In summary, the range of weakness compared to healthy participants increased with age, so DMD patients aged 80 months achieved 79% of normal grip strength while older participants achieved 28%. The bulb dynamometer was an efficient instrument to detect the progression of muscular weakness in DMD participants. In despite of the advance of weakness, functional performance measured by the Vignos scores remained virtually unchanged.
